# Selection of Medical Diagnostic Codes for Analysis of Electronic Patient Records. Application to Stroke in a Primary Care Database

**DOI:** 10.1371/journal.pone.0007168

**Published:** 2009-09-24

**Authors:** Martin C. Gulliford, Judith Charlton, Mark Ashworth, Anthony G. Rudd, Andre Michael Toschke, for the eCRT Research Team

**Affiliations:** Division of Health and Social Care Research, King's College London, London, United Kingdom; Albert Einstein College of Medicine, United States of America

## Abstract

**Background:**

Electronic patient records from primary care databases are increasingly used in public health and health services research but methods used to identify cases with disease are not well described. This study aimed to evaluate the relevance of different codes for the identification of acute stroke in a primary care database, and to evaluate trends in the use of different codes over time.

**Methods:**

Data were obtained from the General Practice Research Database from 1997 to 2006. All subjects had a minimum of 24 months of up-to-standard record before the first recorded stroke diagnosis. Initially, we identified stroke cases using a supplemented version of the set of codes for prevalent stroke used by the Office for National Statistics in *Key health statistics from general practice 1998* (ONS codes). The ONS codes were then independently reviewed by four raters and a restricted set of 121 codes for ‘acute stroke’ was identified but the kappa statistic was low at 0.23.

**Results:**

Initial extraction of data using the ONS codes gave 48,239 cases of stroke from 1997 to 2006. Application of the restricted set of codes reduced this to 39,424 cases. There were 2,288 cases whose index medical codes were for ‘stroke annual review’ and 3,112 for ‘stroke monitoring’. The frequency of stroke review and monitoring codes as index codes increased from 9 per year in 1997 to 1,612 in 2004, 1,530 in 2005 and 1,424 in 2006. The one year mortality of cases with the restricted set of codes was 29.1% but for ‘stroke annual review,’ 4.6% and for ‘stroke monitoring codes’, 5.7%.

**Conclusion:**

In the analysis of electronic patient records, different medical codes for a single condition may have varying clinical and prognostic significance; utilisation of different medical codes may change over time; researchers with differing clinical or epidemiological experience may have differing interpretations of the relevance of particular codes. There is a need for greater transparency in the selection of sets of codes for different conditions, for the reporting of sensitivity analyses using different sets of codes, as well as sharing of code sets among researchers.

## Introduction

Electronic patient records from primary care databases are increasingly used in public health and health services research with applications in disease epidemiology, drug utilisation and pharmacoepidemiology [1]. Identification of patients with specific diseases for study from electronic patient records typically requires the use of selected medical codes but the process of selecting codes has not been well described. In an early publication from the General Practice Research Database (GPRD), the Office for National Statistics (ONS) published sets of codes for a range of diseases and conditions included in the publication Key *Health Statistics from General Practice* [2]. This example has not often been followed by researchers, even though the selection of appropriate sets of medical codes is recognised to be a difficult yet important component of research using electronic patient records. This paper therefore describes the procedure we used to select a set of codes for diagnosis of stroke in primary care.

Stroke is a long-term condition of public health importance that may be investigated in primary care databases. However, stroke represents an acute illness, around the time of initial occurrence or recurrence, as well as a long-term condition which may require secondary preventive medical care, rehabilitation and supportive care. One of the first studies to report on the epidemiology of stroke in a primary care database was the UK Office for National Statistics report Key *Health Statistics in General Practice* [2]. This publication reported on the prevalence of stroke in the UK General Practice Research Database (GPRD). Subjects with stroke were identified using a set of 192 medical codes that included diagnostic codes for acute stroke, as well as prevalent stroke and cerebrovascular disease. In a GPRD-based study we used the ONS codes with a 2 year stroke code free run-in period to identify incident stroke events [3]. However, in a study of trends in case fatality, we identified important trends in the use of different medical codes for stroke over time. In the present analysis, therefore, we aimed to re-evaluate the set of codes described by ONS to determine whether a subset of codes for acute stroke could be identified. The methods used for evaluation included: i) reclassification of codes by four independent raters; ii) evaluation of trends over time in the use of medical codes for stroke; and iii) evaluation of one year mortality for groups of subjects with different index medical codes for stroke. We also aimed to evaluate trends over time in the utilisation of different codes.

## Methods

### General Practice Research Database

The General Practice Research Database (GPRD) is a large database comprising the electronic patient records from approximately 5% of UK general practices. A number of studies have evaluated the validity of diagnoses recorded in GPRD with generally satisfactory results [4], [5]. Data were collected into the GPRD from 1987 up to the present. Clinical entries in the GPRD were coded initially using the Oxford Medical Information Systems (OXMIS) codes and later using READ codes, with the date of transition from Oxmis to READ coding systems varying for different practices in the database [1].

### Data extraction

Initially, we used the set of diagnostic codes for prevalent stroke that was used by the Office for National Statistics in the publication Key *Health Statistics from General Practice* [2].

This set of codes does not include transient ischaemic attacks. There are 192 codes in the set used by ONS to define prevalent stroke. These were supplemented with codes from the READ and GPRD medical code dictionaries, giving a set of 202 codes. These codes were used for initial data extraction.

The baseline population consisted of all up-to-standard patients (UTS) in GPRD from 1st January 1987 to 31st December 2006 inclusive. In the GPRD data are classified as ‘up-to-standard’ when they are judged to be of a high enough quality to be used for research. Cases were all patients with a first recorded stroke code during the period 1st January 1997 to 31st December 2006 inclusive. The date of stroke diagnosis was defined as the first date on which a stroke medical code was recorded. Cases were selected if they had at least 24 months of up-to-standard follow-up, prior to the date of diagnosis of stroke. This process yielded a sample of 48,239 subjects.

### Data analysis

Three methods were used to evaluate the medical codes and the extracted data.

#### i) reclassification of codes by four independent observers

The list of 202 Read and Oxmis codes was independently reviewed by four different medically qualified raters. These included a public health/health services researcher, an epidemiologist, a stroke physician and a general practitioner. Each rater reviewed each code and determined, based on their clinical judgement, whether use of the code was likely to indicate an acute stroke event. Raters were compared using kappa statistics. For each code, the ratings were summed to give a score ranging from zero, with no raters agreeing that the code indicated acute stroke, to four, with all raters agreeing that the code was consistent with acute stroke. Based on consensus, codes rated 3 or 4 were classed as ‘acute stroke’. A rating of three affirmatives was used as a cut-point because one rater did not classify subarachnoid haemorrhage as stroke. Codes that were excluded through this process included codes for long-term stroke management including sequelae of stroke, extracranial atherosclerotic disease, disorders of the intracranial venous sinuses and other conditions. Three codes for stroke annual review or stroke monitoring were also classified into stroke annual review codes, including “Stroke/CVA annual review” (662e.00), and stroke monitoring codes, including “stroke monitoring” (662M.00) and “haemorrhagic stroke monitoring” (662o.00).

#### ii) evaluation of trends over time in the use of medical codes for stroke

We evaluated trends over time in the use of different sets of codes including those affirmed by 0, 1, 2, 3 or 4 raters. We analysed the medical codes recorded on the stroke index date, that is the date of the earliest reported stroke code in the medical record. Where more than one stroke code was recorded on the index date then the code with the highest rating was selected for analysis. We also used the sets of codes resulting from reclassification – ‘acute stroke’, ‘stroke annual review’ and ‘stroke monitoring’. We estimated incidence rates for stroke for different groups of codes. Rates were estimated by study year, standardising by age and sex to the European Standard Population for reference.

#### iii) evaluation of one year mortality for groups of subjects with different types of first medical code for stroke

We compared mortality within one year of stroke incidence for the same sets of codes outlined above. One year case fatality was estimated from a failure function in order to allow for censoring of cases that transferred out or reached the end of the study before one year follow-up was completed.

### Ethics

The study was implemented utilising an anonymised dataset from the General Practice Research Database. The study protocol was approved by the Independent Scientific Advisory Committee (ISAC) of the Medicines and Healthcare products Regulatory Agency (MHRA) (ISAC Protocol No. 07_027R).

## Results

### Review of stroke codes

When the ONS codes were rated by four reviewers there was poor agreement concerning whether the codes were consistent with acute stroke. Table 1 shows pairwise values for percent agreement and kappa. While there was moderate or good agreement between raters one and three, one and four and between two and three, there was only weak agreement between raters one and two, two and four and three and four (Table 1). The value for kappa for all four raters was low at 0.23 suggesting no overall agreement. However, more than 80% of new stroke events were associated with codes that either three or four raters agreed were consistent with acute stroke. The number of raters who indicated a code was consistent with acute stroke and numbers of codes, were as follows: zero affirmative ratings, four codes; one affirmative rating, 36 codes; two affirmatives, 41 codes; three affirmatives, 41 codes; four affirmatives, 80 codes. Thus each of the raters agreed that codes for cerebral infarction, cerebral haemorrhage, cerebral embolism and ‘cerebral vascular accident’ were consistent with acute stroke. Of the 41 codes that only three raters affirmed as consistent with acute stroke, 16 were for subarachnoid haemorrhage which is included in the WHO definition of stroke but not in the definition of stroke used in the Quality and Outcomes Framework of the UK general practice contract. A further 12 codes were for hemiplegia or paralysis, which is a common presenting feature of acute stroke. Of the codes which two or fewer raters considered to be indicative of acute stroke, the majority of codes were for intracranial or extracranial arterial disease without mention of infarction, diseases of the cerebral venous sinuses, or for sequelae of stroke. Raters showed considerable disagreement over these codes. Based on these findings, we considered codes that were affirmed by three or more raters were consistent with acute stroke. This set of codes for ‘acute stroke’ included 121 codes.

**Table 1:** Agreement between four raters.

	Rater 1 Agreement %	Rater 1 Kappa	Rater 2 Agreement %	Rater 2 Kappa	Rater 3 Agreement %	Rater 3 Kappa
**Rater 2**	64.4	0.12	-	-	-	-
**Rater 3**	76.2	0.42	76.2	0.30	-	-
**Rater 4**	77.7	0.54	48.0	−0.10	61.9	0.20

Figures are observed percent agreement and kappa statistic for independent rating of 202 codes by each of four raters.

### Utilisation of stroke codes

Initial extraction of data using the ONS codes gave 48,239 cases of stroke from 1997 to 2006. The 48,239 subjects had a total of 70,288 stroke medical codes recorded on the stroke index date. The codes used and their relative frequencies are shown in the Table 2. There were only 17 codes that each accounted for more than 1% of subjects, and just two codes accounted for nearly half of subjects.

**Table 2:** READ and OXMIS terms for stroke, results of rating for acute stroke and relative frequency among 70,288 index stroke codes recorded in 48,239 subjects.

Type	Code	Term	Sum of 4 ratings	Frequency in 70,288 index codes	Percent of index codes
READ	G66..00	Stroke and cerebrovascular accident unspecified	4	21354	30.38
READ	G66..11	CVA unspecified	4	15426	21.95
OXMIS	4369A	CVA (cerebrovascular accident)	4	2859	4.07
READ	G66..13	CVA - Cerebrovascular accident unspecified	4	2254	3.21
READ	G64z.00	Cerebral infarction NOS	4	2224	3.16
READ	G61..11	CVA - cerebrovascular accid due to intracerebral haemorrhage	4	1334	1.90
READ	G61..00	Intracerebral haemorrhage	4	1251	1.78
READ	G64z.12	Cerebellar infarction	4	762	1.08
OXMIS	4369B	Stroke	4	525	0.75
READ	G64..13	Stroke due to cerebral arterial occlusion	4	472	0.67
READ	G640.00	Cerebral thrombosis	4	402	0.57
READ	G667.00	Left sided CVA	4	393	0.56
READ	G64..12	Infarction - cerebral	4	351	0.50
READ	G668.00	Right sided CVA	4	302	0.43
READ	G61..12	Stroke due to intracerebral haemorrhage	4	258	0.37
READ	G66..12	Stroke unspecified	4	205	0.29
READ	G664.00	Cerebellar stroke syndrome	4	200	0.28
READ	G663.00	Brain stem stroke syndrome	4	190	0.27
READ	G613.00	Cerebellar haemorrhage	4	179	0.25
READ	G64z300	Right sided cerebral infarction	4	132	0.19
READ	G64z200	Left sided cerebral infarction	4	117	0.17
READ	G641.00	Cerebral embolism	4	97	0.14
READ	G61z.00	Intracerebral haemorrhage NOS	4	93	0.13
READ	G63y000	Cerebral infarct due to thrombosis of precerebral arteries	4	62	0.09
READ	G64z000	Brainstem infarction	4	61	0.09
READ	G640000	Cerebral infarction due to thrombosis of cerebral arteries	4	48	0.07
OXMIS	3479AG	Cerebellar infarction	4	42	0.06
READ	G6X..00	Cerebrl infarctn due/unspcf occlusn or sten/cerebrl artrs	4	41	0.06
READ	G64z400	Infarction of basal ganglia	4	40	0.06
READ	G665.00	Pure motor lacunar syndrome	4	32	0.05
READ	G63y100	Cerebral infarction due to embolism of precerebral arteries	4	31	0.04
READ	G614.00	Pontine haemorrhage	4	28	0.04
READ	G62z.00	Intracranial haemorrhage NOS	4	22	0.03
OXMIS	4339NF	Infarct cerebral	4	17	0.02
READ	G61X000	Left sided intracerebral haemorrhage, unspecified	4	17	0.02
READ	G61X100	Right sided intracerebral haemorrhage, unspecified	4	17	0.02
OXMIS	4319CE	Haemorrhage intracerebral	4	15	0.02
READ	G6W..00	Cereb infarct due unsp occlus/stenos precerebr arteries	4	14	0.02
READ	G611.00	Internal capsule haemorrhage	4	13	0.02
READ	G641000	Cerebral infarction due to embolism of cerebral arteries	4	11	0.02
OXMIS	4319	Haemorrhage cerebral	4	10	0.01
READ	G676000	Cereb infarct due cerebral venous thrombosis, nonpyogenic	4	10	0.01
READ	G666.00	Pure sensory lacunar syndrome	4	10	0.01
READ	G62..00	Other and unspecified intracranial haemorrhage	4	8	0.01
OXMIS	4339PL	Parietal lobe infarct	4	7	0.01
READ	G64z.11	Brainstem infarction NOS	4	5	0.01
READ	Gyu6400	[X]Other cerebral infarction	4	5	0.01
OXMIS	4369AR	Cerebrovascular accident right	4	4	0.01
OXMIS	4339FL	Infarct frontal lobe	4	3	0.00
OXMIS	4369AL	Cerebrovascular accident left	4	3	0.00
OXMIS	4339BT	Infarct brain stem	4	3	0.00
READ	G641.11	Cerebral embolus	4	3	0.00
READ	G610.00	Cortical haemorrhage	4	3	0.00
OXMIS	4349	Embolism cerebral	4	2	0.00
OXMIS	4339TL	Temporal lobe infarct	4	2	0.00
READ	G612.00	Basal nucleus haemorrhage	4	2	0.00
READ	G616.00	External capsule haemorrhage	4	2	0.00
OXMIS	4339WL	Wallenberg's syndrome	4	2	0.00
READ	G63..11	Infarction - precerebral	4	2	0.00
READ	Gyu6200	[X]Other intracerebral haemorrhage	4	2	0.00
OXMIS	4319NA	Haematoma brain nontraumatic	4	1	0.00
OXMIS	4319NG	Haemorrhage brain nontraumatic	4	1	0.00
OXMIS	4339PP	Infarcts cerebral septic	4	1	0.00
OXMIS	4339	Thrombosis cerebral	4	1	0.00
READ	G61X.00	Intracerebral haemorrhage in hemisphere, unspecified	4	1	0.00
OXMIS	4369CR	Accident cerebral	4	0	0.00
OXMIS	4370	Cerebral ischaemia hypertensive	4	0	0.00
OXMIS	4360A	Cerebrovascular accident with hypertensi	4	0	0.00
OXMIS	4340	Embolism cerebral with hypertension	4	0	0.00
OXMIS	4340CR	Embolism intracranial with hypertension	4	0	0.00
OXMIS	4310	Haemorrhage intracerebral with hypertens	4	0	0.00
OXMIS	344 BF	Hemiplegia flaccid	4	0	0.00
OXMIS	4360HP	Hypertensive hemiplegia	4	0	0.00
OXMIS	4339P	Pontine infarct	4	0	0.00
OXMIS	4360B	Stroke with hypertension	4	0	0.00
OXMIS	4369BN	Syndrome stroke	4	0	0.00
READ	G618.00	Intracerebral haemorrhage, multiple localized	4	0	0.00
READ	Gyu6G00	[X]Cereb infarct due unsp occlus/stenos precerebr arteries	4	0	0.00
READ	Gyu6300	[X]Cerebrl infarctn due/unspcf occlusn or sten/cerebrl artrs	4	0	0.00
READ	Gyu6F00	[X]Intracerebral haemorrhage in hemisphere, unspecified	4	0	0.00
READ	G64..11	CVA - cerebral artery occlusion	3	2384	3.39
READ	G60..00	Subarachnoid haemorrhage	3	2101	2.99
READ	F22..11	Hemiparesis	3	1749	2.49
READ	F22..00	Hemiplegia	3	830	1.18
READ	F22z.00	Hemiplegia NOS	3	175	0.25
OXMIS	4309	Subarachnoid haemorrhage	3	147	0.21
READ	F222.00	Left hemiplegia	3	96	0.14
READ	F223.00	Right hemiplegia	3	92	0.13
READ	G600.00	Ruptured berry aneurysm	3	43	0.06
READ	G602.00	Subarachnoid haemorrhage from middle cerebral artery	3	42	0.06
READ	2833	O/E - hemiplegia	3	37	0.05
READ	G60X.00	Subarachnoid haemorrh from intracranial artery, unspecif	3	34	0.05
READ	G617.00	Intracerebral haemorrhage, intraventricular	3	32	0.05
READ	G60z.00	Subarachnoid haemorrhage NOS	3	30	0.04
READ	G64z111	Lateral medullary syndrome	3	18	0.03
OXMIS	344 BL	Hemiplegia left	3	17	0.02
OXMIS	4369VA	Vascular accident	3	10	0.01
OXMIS	344 BR	Hemiplegia right	3	10	0.01
READ	G604.00	Subarachnoid haemorrhage from posterior communicating artery	3	9	0.01
OXMIS	4319CR	Haemorrhage intracranial	3	8	0.01
READ	G603.00	Subarachnoid haemorrhage from anterior communicating artery	3	8	0.01
READ	G605.00	Subarachnoid haemorrhage from basilar artery	3	7	0.01
OXMIS	4380	Cerebrovascular disease with hypertensio	3	5	0.01
OXMIS	4379	Ischaemia cerebral	3	5	0.01
OXMIS	344 HF	Hemiparesis facial	3	2	0.00
OXMIS	4330	Cerebral thrombosis with hypertension	3	2	0.00
OXMIS	4359CC	Caroticovertebral ischaemia	3	1	0.00
OXMIS	4349CR	Embolism intracranial	3	1	0.00
READ	G615.00	Bulbar haemorrhage	3	1	0.00
READ	G606.00	Subarachnoid haemorrhage from vertebral artery	3	1	0.00
READ	Gyu6100	[X]Other subarachnoid haemorrhage	3	1	0.00
OXMIS	4339BC	Clot blood brain	3	0	0.00
OXMIS	4380HP	Hemiplegia with hypertension	3	0	0.00
OXMIS	344	Paralysis cerebral	3	0	0.00
OXMIS	344 B	Paralysis hemiplegia	3	0	0.00
OXMIS	4359PR	Prolonged residual ischaemic neuro defec	3	0	0.00
OXMIS	4300	Subarachnoid haemorrhage with hypertensi	3	0	0.00
OXMIS	4339WA	Thrombosis posterior cerebellar artery	3	0	0.00
READ	G601.00	Subarachnoid haemorrhage from carotid siphon and bifurcation	3	0	0.00
READ	Gyu6E00	[X]Subarachnoid haemorrh from intracranial artery, unspecif	3	0	0.00
READ	Gyu6000	[X]Subarachnoid haemorrhage from other intracranial arteries	3	0	0.00
READ	14A7.11	H/o: CVA	2	770	1.10
READ	14A7.12	H/O: stroke	2	755	1.07
OXMIS	4389	Cerebrovascular disease	2	168	0.24
READ	14A7.00	H/O: CVA/stroke	2	70	0.10
READ	G68X.00	Sequelae of stroke,not specfd as h'morrhage or infarction	2	54	0.08
READ	F221.00	Spastic hemiplegia	2	31	0.04
READ	F053.00	Thrombophlebitis of central nervous system venous sinuses	2	16	0.02
READ	F051.00	Thrombosis of central nervous system venous sinuses	2	14	0.02
READ	G620.00	Extradural haemorrhage - nontraumatic	2	13	0.02
READ	G630.00	Basilar artery occlusion	2	12	0.02
READ	G683.00	Sequelae of cerebral infarction	2	12	0.02
READ	F220.00	Flaccid hemiplegia	2	12	0.02
OXMIS	442 CA	Aneurysm cerebral artery	2	8	0.01
READ	F051000	Thrombosis cavernous sinus	2	7	0.01
READ	G631.12	Thrombosis, carotid artery	2	7	0.01
READ	F051300	Thrombosis transverse sinus	2	6	0.01
READ	F051200	Thrombosis lateral sinus	2	4	0.01
READ	F050000	Embolism cavernous sinus	2	3	0.00
READ	662o.00	Haemorrhagic stroke monitoring	2	3	0.00
READ	G681.00	Sequelae of intracerebral haemorrhage	2	2	0.00
READ	F051z00	Thrombosis of central nervous system venous sinus NOS	2	2	0.00
OXMIS	442 C	Cerebral arteriovenous aneurysm	2	1	0.00
READ	F050100	Embolism superior longitudinal sinus	2	1	0.00
READ	G63y.00	Other precerebral artery occlusion	2	1	0.00
OXMIS	4379AM	Aneurysm cerebrovascular arterioscleroti	2	0	0.00
OXMIS	366 CT	Choroidal thrombosis	2	0	0.00
OXMIS	4309M	Meningeal haemorrhage	2	0	0.00
OXMIS	4389PP	Poststroke paralysis	2	0	0.00
OXMIS	4389PN	Syndrome poststroke	2	0	0.00
OXMIS	4379TA	Thromboangiitis cerebral	2	0	0.00
OXMIS	4329T	Thrombosis carotid artery	2	0	0.00
OXMIS	321 CV	Thrombosis cavernous sinus	2	0	0.00
OXMIS	321 LT	Thrombosis lateral sinus	2	0	0.00
OXMIS	321 TL	Thrombosis superior sagittal sinus	2	0	0.00
READ	F050z00	Embolism central nervous system venous sinus NOS	2	0	0.00
READ	F050200	Embolism lateral sinus	2	0	0.00
READ	F050.00	Embolism of central nervous system venous sinus	2	0	0.00
READ	F050300	Embolism transverse sinus	2	0	0.00
READ	F050.11	Embolus of central nervous system venous sinus	2	0	0.00
READ	F051100	Thrombosis of superior longitudinal sinus	2	0	0.00
READ	Gyu6C00	[X]Sequelae of stroke,not specfd as h'morrhage or infarction	2	0	0.00
READ	662M.00	Stroke monitoring	1	3145	4.47
READ	662e.00	Stroke/CVA annual review	1	2345	3.34
READ	G64..00	Cerebral arterial occlusion	1	1853	2.64
READ	F26y000	Hemiplegic migraine	1	493	0.70
READ	G621.00	Subdural haemorrhage - nontraumatic	1	211	0.30
READ	G631.00	Carotid artery occlusion	1	154	0.22
OXMIS	3460MH	Migraine hemiplegic	1	57	0.08
OXMIS	4319LN	Subdural haematoma/haemorrhage nontrauma	1	49	0.07
READ	G671z00	Generalised ischaemic cerebrovascular disease NOS	1	17	0.02
READ	G63..00	Precerebral arterial occlusion	1	11	0.02
READ	G632.00	Vertebral artery occlusion	1	10	0.01
READ	G65z100	Intermittent cerebral ischaemia	1	7	0.01
READ	G677300	Occlusion and stenosis of cerebellar arteries	1	4	0.01
READ	G677000	Occlusion and stenosis of middle cerebral artery	1	4	0.01
READ	F05..00	Phlebitis and thrombophlebitis of intracranial sinuses	1	3	0.00
OXMIS	3479C	Cerebral anoxia	1	2	0.00
OXMIS	344 SH	Spastic hemiplegia	1	2	0.00
READ	G65z000	Impending cerebral ischaemia	1	2	0.00
READ	G677200	Occlusion and stenosis of posterior cerebral artery	1	2	0.00
READ	14AK.00	H/O: Stroke in last year	1	1	0.00
READ	G680.00	Sequelae of subarachnoid haemorrhage	1	1	0.00
OXMIS	4379AN	Cerebral aneurysm arteriosclerotic	1	0	0.00
OXMIS	7876PH	Hemiplegic gait	1	0	0.00
OXMIS	4389PL	Late effects CVA	1	0	0.00
OXMIS	321 CE	Thrombosis cerebral sinus	1	0	0.00
READ	G671000	Acute cerebrovascular insufficiency NOS	1	0	0.00
READ	G633.00	Multiple and bilateral precerebral arterial occlusion	1	0	0.00
READ	G677100	Occlusion and stenosis of anterior cerebral artery	1	0	0.00
READ	G677400	Occlusion+stenosis of multiple and bilat cerebral arteries	1	0	0.00
READ	F052z00	Phlebitis of central nervous system venous sinus NOS	1	0	0.00
READ	F052.00	Phlebitis of central nervous system venous sinuses	1	0	0.00
READ	G63z.00	Precerebral artery occlusion NOS	1	0	0.00
READ	F053z00	Thrombophlebitis of central nervous system venous sinus NOS	1	0	0.00
READ	Gyu6600	[X]Occlusion and stenosis of other cerebral arteries	1	0	0.00
READ	Gyu6500	[X]Occlusion and stenosis of other precerebral arteries	1	0	0.00
READ	Gyu6B00	[X]Sequelae of other nontraumatic intracranial haemorrhage	1	0	0.00
READ	E030400	Acute confusional state, of cerebrovascular origin	0	34	0.05
READ	E031400	Subacute confusional state, of cerebrovascular origin	0	23	0.03
READ	G677.00	Occlusion/stenosis cerebral arts not result cerebral infarct	0	4	0.01
OXMIS	344 BT	TRANSIENT HEMIPLEGIA	0	0	0.00

(There were 19,497 subjects with two codes recorded on the index date and 2,552 subjects with three or more codes recorded on the index date)

The distribution of different sets of codes, as index codes, within this sample is shown in Table 3. Codes that received zero affirmative ratings were rarely utilised as index codes accounting for 56 (0.1%) subjects out of the entire sample. Codes that received one affirmative rating accounted for 14.7% of the patient sample, but the number of cases identified through these codes increased from 121 per year in 1997 to 1,861 in 2004, 1,730 in 2005, and 1,618 in 2006. Of 7,091 first occurrences of these codes 5,209 (73%) were in 2004 or later. This increase was accounted for by increased utilisation of codes for stroke annual review or stroke monitoring. Overall, there were 3,112 (6.5%) of cases initially diagnosed through codes for ‘stroke monitoring’ and 2,288 (4.7%) diagnosed through codes for ‘stroke annual review’. Codes affirmed by two raters accounted for 3.5% of the patient sample. Codes that received three or four affirmative ratings accounted for 81.7% of the patient sample. However, these codes accounted for 93% of the patient sample in 1997 but 68% in 2006. There were a further 860 subjects with valid acute stroke codes ever recorded, affirmed by three or four raters, in whom the valid acute stroke code was not an index code. There were 293/860 cases in whom the valid acute stroke code was recorded within 30 days of the index stroke code.

**Table 3:** Number of new stroke cases by study year and type of codes used for case selection.

	ONS codes (202)	Zero affirmatives (4)	One affirmative (36)	Two affirmatives (41)	Three affirmatives (41)	Four affirmatives (80)	‘Stroke monitoring’ (2)	‘Stroke annual review’ (1)	‘Acute stroke’ (121)
**1997**	3,403	3	121	127	340	2,812	9	0	3,152
**1998**	3,812	2	153	156	412	3,089	19	1	3,501
**1999**	4,065	2	157	167	433	3,306	37	4	3,739
**2000**	4,228	6	216	151	467	3,388	53	1	3,855
**2001**	4,706	12	219	200	512	3,763	86	5	4,275
**2002**	5,042	5	350	201	554	3,932	185	5	4,486
**2003**	5,371	10	666	201	682	3,812	384	45	4,494
**2004**	6,307	8	1,861	168	749	3,521	885	727	4,270
**2005**	5,853	4	1,730	149	688	3,282	701	829	3,970
**2006**	5,452	4	1,618	148	580	3,102	753	671	3,682
**Total**	48,239	56	7,091	1,668	5,417	34,007	3,112	2,288	39,424
**Row %**		0.1	14.7	3.5	11.2	70.5	6.5	4.7	81.7

Figures in parentheses are numbers of codes in set.

Table 4 shows age and sex standardised incidence rates for stroke by study year based on different sets of codes. The incidence of stroke using all codes remained approximately constant during the earlier part of this study period. However, the EU-standardised incidence of stroke increased from 1.13 per 1,000 in 2002 to 1.19 in 2003 and 1.36 in 2004 before declining to 1.15 in 2006. The incidence of stroke associated with stroke monitoring and stroke annual review codes showed a large increase over the period, with a steep increase between 2002 and 2004. The incidence of ‘acute stroke’ remained approximately constant initially but later declined from 1.00 per 1,000 in 2003 to 0.76 in 2006.

**Table 4:** Incidence of first stroke by study year and type of codes used for case selection.

	ONS codes (202)	Zero affirmatives (4)	One affirmative (36)	Two affirmatives (41)	Three affirmatives (41)	Four affirmatives (80)	‘Stroke monitoring’ (2)	‘Stroke annual review’ (1)	‘Acute stroke’ (121)
**1997**	1.12	0.00	0.05	0.04	0.14	0.89	0.00	0.00	1.03
**1998**	1.16	0.00	0.06	0.05	0.15	0.91	0.01	0.00	1.05
**1999**	1.10	0.00	0.05	0.05	0.14	0.86	0.01	0.00	1.00
**2000**	1.05	0.00	0.06	0.04	0.14	0.81	0.01	0.00	0.95
**2001**	1.08	0.00	0.06	0.04	0.14	0.83	0.02	0.00	0.97
**2002**	1.13	0.00	0.09	0.05	0.15	0.85	0.04	0.00	1.00
**2003**	1.19	0.00	0.16	0.04	0.18	0.81	0.08	0.01	1.00
**2004**	1.36	0.00	0.41	0.04	0.19	0.73	0.19	0.16	0.91
**2005**	1.23	0.00	0.37	0.03	0.17	0.66	0.15	0.17	0.83
**2006**	1.15	0.00	0.35	0.03	0.14	0.63	0.16	0.14	0.76

Figures are rates per 1,000 years standardised by age and sex to the European standard population.

Table 5 shows the one year case fatality of groups of subjects with different index codes. Data for 394 cases in which the first stroke code was after the death date were excluded. The mortality of 47,845 subjects with stroke identified using the ONS codes was 25.5% within one year following the initial stroke event. The case fatality of subjects classified as having ‘acute stroke’ was 29.1%. The case fatality of subjects identified through ‘stroke annual review’ codes was 4.6% and for ‘stroke monitoring’ codes 5.7%. Codes that were not rated in the affirmative by any rater were infrequently utilised but were associated with unexpectedly high mortality. The reasons for this finding are unclear.

**Table 5:** One year mortality by type of codes used for stroke selection.

	Number	Deaths within 12 months	One year case fatality^a^ (%) (95% confidence interval)
**ONS codes**	47,845	11,876	25.5 (25.1 to 25.9)
**Zero affirmatives**	56	23	43.1 (31.1 to 57.5)
**One affirmative**	7,073	405	6.2 (5.6 to 6.8)
**Two affirmatives**	1,666	362	22.6 (20.6 to 24.7)
**Three affirmatives**	5,369	1,218	23.3 (22.1 to 24.4)
**Four affirmatives**	33,681	9,868	30.0 (29.5 to 30.5)
**‘Stroke monitoring’**	3,105	159	5.7 (4.9 to 6.6)
**‘Stroke annual review’**		94	4.6 (3.8 to 5.7)
**‘Acute stroke’**	39,050	11,086	29.1 (28.6 to 29.6)

a estimated from failure function allowing for censoring.

## Discussion

### Main findings

This paper has presented an analysis of the codes for prevalent stroke used by the Office for National Statistics. Our initial approach to the evaluation of strokes in GPRD was to identify subjects with a first-ever recorded code from the ONS set, with at least a period of 24 stroke record free months available before the index diagnosis. This study yielded plausible results consistent with the literature. However, the ONS code set was designed to identify cases with prevalent stroke. In order to identify a possible set of codes for acute stroke, we used a combination of methods. Although the raters disagreed concerning the significance of codes that were not clearly associated with cerebral haemorrhage or infarction, by restricting attention to those codes for which three or four raters agreed that acute stroke was probable, we were able to achieve consensus concerning the selection of codes. Within the set of codes for ‘acute stroke’ there was disagreement concerning the inclusion of subarachnoid haemorrhage but this is compatible with inconsistent definitions that are in clinical use. The Quality and Outcomes Framework (QOF) for the UK General Practice contract does not include subarachnoid haemorrhage [6] and this was reflected in the rating performed by the general practitioner. Read codes eligible for QOF include those for cerebral haemorrhage, cerebral infarction, cerebral embolism as well as stroke and ‘CVA’ (Cerebral Vascular Accident). All QOF eligible codes were included within the set of codes for ‘acute stroke’ in this research, supporting the validity of the latter.

There have evidently been major selective increases in the use of specific codes subsequent to the introduction of the QOF. The use of codes for stroke annual review and stroke monitoring has greatly increased in frequency and peaked shortly after the inclusion in the QOF with a subsequent plateau. These codes were sometimes used as incident stroke codes, with no previous mention of stroke in at least the preceding 24 months. Cases identified in this way were associated with low mortality. If associated with acute stroke events they might represent mild cases possibly explaining the low mortality. Unfortunately, we cannot directly examine if the use of codes might be associated with the severity of the stroke event due to lack of information on stroke severity. However, subjects with stroke may be admitted directly to hospital and their first contact with the general practice may be for review at some later date. The stroke onset date may therefore be imprecisely recorded in primary care, perhaps often being recorded after the true stroke onset date. A potential bias in the recording of stroke dates might also explain the low mortality among patients with those codes as first stroke codes. Additional validation of stroke diagnosis dates through analysis of information on prescribing, utilisation of secondary care services or test results may be important when the precise diagnosis dates are required. The use of codes for monitoring and review may be compatible with the analysis of certain aspects of stroke research in the general practice record since a re-analysis of our earlier research excluding these codes and only considering the 121 codes identified in this paper did not yield different results [3]. Nevertheless, it will be advisable to be cautious in utilising these codes for studies of stroke prognosis and especially for studies of secular trends in stroke outcomes.

### Comparison with other studies

Several studies in the General Practice Research Database (GPRD) have evaluated the epidemiology of stroke [2], [7]–[16]. Analytical studies in GPRD that employed stroke occurrence as an outcome have generally adopted a more restrictive approach to definition. An incident stroke is usually defined as one that is associated with the first-ever recorded stroke code. A minimum period of study time free of recorded stroke codes, usually between one and three years, is often used to precede the first stroke record. Some studies have also excluded subjects ever diagnosed with multiple sclerosis or cancer, as stroke may be diagnosed with less certainty in these subjects [7], [8]. Some studies have also used evidence of relevant health care utilisation including hospital admissions, diagnostic tests and drug prescribing as contributing to the confirmation of stroke diagnoses [7], [8]. These latter approaches are demanding to implement and results may be dependent on subjects gaining access to specialist care, as well as there being adequate recording of relevant measures within the primary care record as well as the specialist record. Studies of stroke epidemiology, requiring the analysis of large numbers of stroke records require approaches that can be more readily implemented.

### Strengths and limitations

Our study has the strengths of a very large sample that was drawn from a large number of UK general practices. In common with all studies that use electronic patient records, the data are subject to variation in clinical practice, as well as clinical uncertainty in diagnosis. There will always be a proportion of cases in whom the diagnosis may be uncertain. We utilised four independent raters with differing specialist expertise to evaluate diagnostic codes. We also evaluated different sets of codes for their impact on stroke incidence and stroke case fatality. A limitation of this research is the lack of a reference method to establish stroke diagnoses. This would require evaluation of electronic records against clinical records, with access to specialist records for details of scan results in most cases. In spite of this limitation we believe that the methods adopted in this study may provide a useful approach to the selection of codes for analysis of electronic patient records in future studies.

### Conclusions

This study raises several questions concerning the selection of medical codes to provide case definitions in research that utilises electronic patient records. Firstly, utilisation of different medical codes may change over time. Secondly, different medical codes for a single condition may have varying clinical and prognostic significance. Thirdly, researchers with differing clinical or epidemiological experience may have differing interpretations of the relevance of particular codes. Since these issues may sometimes have an impact on the results of a study, there is a need for greater transparency in the selection of sets of codes for different conditions, for the reporting of sensitivity analyses using different sets of codes, as well as sharing of code sets among researchers.
